# Extreme Sexual Brain Size Dimorphism in Sticklebacks: A Consequence of the Cognitive Challenges of Sex and Parenting?

**DOI:** 10.1371/journal.pone.0030055

**Published:** 2012-01-19

**Authors:** Alexander Kotrschal, Katja Räsänen, Bjarni K. Kristjánsson, Mike Senn, Niclas Kolm

**Affiliations:** 1 Department of Animal Ecology, Evolutionary Biology Centre, University of Uppsala, Uppsala, Sweden; 2 Department of Aquatic Ecology, Eawag and Institute of Integrative Biology, ETH Zürich, Zürich, Switzerland; 3 Department of Aquaculture and Fish Biology, Hólar University College, Hólar, Iceland; University of Lethbridge, Canada

## Abstract

Selection pressures that act differently on males and females produce numerous differences between the sexes in morphology and behaviour. However, apart from the controversial report that males have slightly heavier brains than females in humans, evidence for substantial sexual dimorphism in brain size is scarce. This apparent sexual uniformity is surprising given that sexually distinct selection pressures are ubiquitous and that brains are one of the most plastic vertebrate organs. Here we demonstrate the highest level of sexual brain size dimorphism ever reported in any vertebrate: male three-spined stickleback of two morphs in an Icelandic lake have 23% heavier brains than females. We suggest that this dramatic sexual size dimorphism is generated by the many cognitively demanding challenges that males are faced in this species, such as an elaborate courtship display, the construction of an ornate nest and a male-only parental care system. However, we consider also alternative explanations for smaller brains in females, such as life-history trade-offs. Our demonstration of unprecedented levels of sexual dimorphism in brain size in the three-spined stickleback implies that behavioural and life-history differences among the sexes can have strong effects also on neural development and proposes new fields of research for understanding brain evolution.

## Introduction

Divergent selection pressures between males and females have produced many differences between the sexes in morphology and behaviour [1]. The mechanisms responsible for these sexual differences include sexual selection, intersexual food competition, and reproductive role division [2]. One example of sexual dimorphism is the allegedly larger brains of men compared to women [3]. However, these findings remain heavily criticized both for unsuitable statistical methods [4], [5] and the inappropriateness of setting up expectations about sexual differences in intelligence. Apart from this questionable example in humans and various species where the sexes differ in their structural architecture of the brain [6], [7], [8], [9], cases of sexual dimorphism in overall brain size are virtually absent. This is surprising considering the generally distinct selection pressures acting on males and females [1], the concurring sex-specific specializations, and the enormous cross-species brain size variation commonly associated with such specializations [10].

Numerous hypotheses exist as to why sexual dimorphism in brain size should evolve and these hypotheses in turn build on that larger brains are generally associated with greater cognitive abilities [11], [12], [13], [14]. Apart from the selection pressures that previous studies have identified to be associated with increased brain mass independent of sex, such as living in complex social groups (the ‘social brain hypothesis’ [15]) or urban environments [16], some selection pressures are likely to impact one sex more than the other. Such sex-specific selection pressures include constructing complex structures involved in sexual displays [17], providing uniparental brood care (the ‘parental brain hypothesis’ [18]), and that the sex under stronger sexual selection should have larger brains [19]. Moreover, brain tissue is very costly to construct and maintain [20], and an increase in neural mass could therefore be associated with a decrease in other costly tissues, such as gut or testis mass [20], [21]. Therefore, existing differences between the sexes in any of these aspects of social behaviours and/or investments into other costly organs are expected to generate sexual dimorphism in brain size.

Here we test whether males and females in three-spined stickleback (*Gasterosteus aculeatus*) differ in brain size, as would be expected if sexually divergent selection pressures generate sexual dimorphism in brain size evolution. Our samples originate from two different populations in lake Mývatn, Iceland, that inhabit ecologically distinct habitats differing in for instance temperature and oxygen levels [22]. This species is well-suited for investigation of sexual brain size dimorphism since both social behaviours and life-history investments differ greatly among the sexes. For instance, males construct elaborate nests, court females intensely during mating and solely provide parental care, whereas females are highly choosy with regards to mate choice [23] and invest heavily in egg production [24]. Moreover, since there are several differences both in life-history traits and in parental care behaviour across the two environments [25], the populations under study offer the opportunity to investigate also the link between neural development and intrinsic factors affected by variation in the physical environment.

## Materials and Methods

We obtained mature stickleback from Lake Mývatn, Iceland [25]. We sampled fish from two sites, which have previously been described as ‘lava’ and ‘mud’ morphs [26], since they inhabit different types of environments and are morphologically [26] and genetically [27] distinct. The lava fish originate from the northeast basin of the lake, where the habitat is characterized by a complex lava structure covered in deep diatom mud, shallow water depth (<0.5 m), stable high temperatures (±23°C due to hydrothermal activity), low oxygen levels (6.3 mg/l) and a lack of vegetation. The north basin is highly productive and has a very high stickleback density [28]. The mud fish originate from the northwest shore of the south basin of the lake, which is characterized by sparse rocks and a fine mud substrate, deeper water (±1.2 m), temperatures that follow the ambient temperatures (ca. ±4°C to 18°C), higher oxygen levels (13 mg/l) and sparse vegetation. The south basin is less productive and has a lower and strongly fluctuating population density [22]. The fish were sampled with minnow traps and brought to Hólar University College, Iceland, where they were kept on an *ad libitum* diet of frozen bloodworms, 24 hours of daylight, and average water temperatures resembling their habitat of origin (lava: ±23°C, mud ca. ±13°C). After 2 months of mating and parental care trials (described in [25]), during which all fish successfully bred, a total of 58 males and 61 females (lava: 27♀, 32♂; mud: 31♀, 28♂) were euthanized with an overdose of phenoxylethanol and placed in 5% buffered paraformaldehyde. The brains were removed and weighed to the nearest mg, body mass was determined to the nearest 0.01 g and standard length (from the tip of the snout to the end of the caudal peduncle) was determined to the nearest 0.1 mm with digital callipers. All dissections and measurements were performed by one person (AK), and done blindly (specimens were identified by a running number).

To control for the effects of brain-to-body allometry, we used log transformation of brain size in conjunction with the inclusion of log body size as a covariate. First, to investigate the effects of sex and habitat on relative brain size, we used an analysis of covariance (ANCOVA) with total brain mass (log transformed) as the dependent variable, sex and habitat as fixed factors, and body size (log-transformed standard length) as a covariate. Second, to investigate between-population differences in males and females separately, we ran two separate ANCOVAs within each sex with habitat as a fixed factor and body size as a covariate. These analyses are suitable since the error rate of body size is expected to be very small compared to the error rate of brain weight [29]. To test for potential body mass differences between the sexes and habitats, which may confound our results, we used an analysis of covariance (ANCOVA) with body mass (log transformed) as the dependent variable, sex and habitat as fixed factors, and body size (log-transformed standard length) as a covariate. All data met the requirements for parametric analyses; all analyses were done with SPSS 19.0 (SPSS Inc., Chicago, IL, USA). The Hólar University College ethical committee approved this study and we adhered to the “Guidelines for the treatment of animals in behavioural research and teaching” published in ‘Animal Behaviour’ 2006, 71, 245–253.

## Results

**Figure 1:**
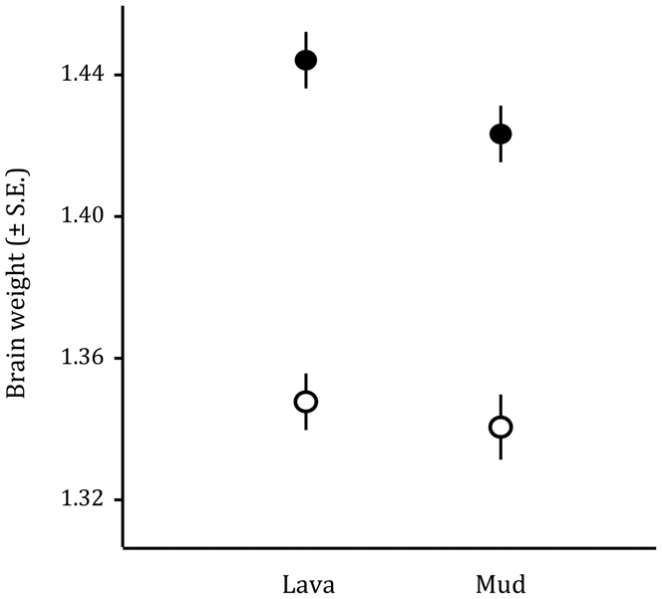
Brain size (g) of male (filled circles) and female (open circles) three-spined sticklebacks from two morphs in Lake Mývatn, Iceland. Depicted are the estimated marginal means from a GLM with (log-transformed) body size as covariate and sex and habitat as factors.

**Figure 2:**
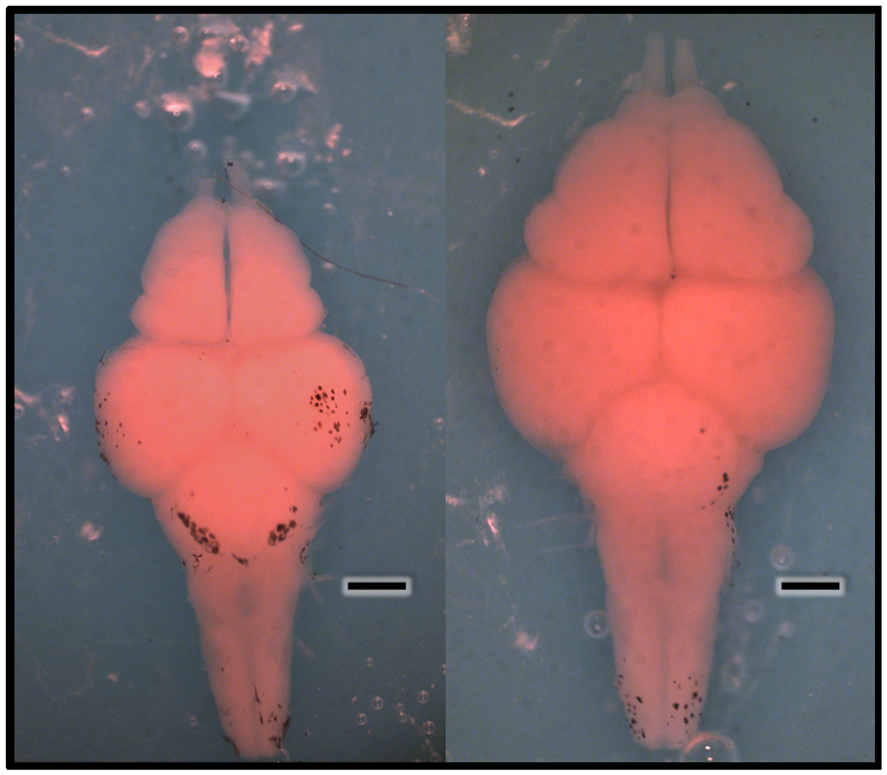
Microscopic image of the dorsal view of a female (left) and a male (right) brain of a three-spined stickleback of 45.0 mm standard length (female body weight: 1.35 g, male weight: 1.33 g). The scales indicate 1 mm.

We found that males had significantly heavier brains than females when controlling for the effect of body size, whereas there were no overall differences in brain mass between mud and lava habitats (ANCOVA: body size: F_1,118_ = 439.50, p<0.0001; sex: F_1,118_ = 112.56, p<0.0001; habitat: F_1,118_ = 2.48, p = 0.118; sex×habitat interaction: F_1,118_ = 0.79, p = 0.376; figure 1). According to our covariate model correcting for body size, male brains were on average 22.8% heavier than female brains for equally sized fish. To visually display the magnitude of this difference, we compared the brains of an average sized male and female (both 45.0 mm in standard length, ♀: 1.35 g, ♂: 1.31 g, figure 2) for which the brains weighed 24.2 mg and 19.7 mg, respectively. When we analysed the sexes separately, males and females differed in the extent of brain size differences between the habitats. While female brain mass did not differ between habitats (ANCOVA: body size: F_1,58_ = 139.64, p<0.0001; habitat: F_1,58_ = 0.003, p = 0.956), males from the lava habitat had significantly heavier brains than males from the mud habitat (ANCOVA: body size: F_1,61_ = 386.31, p<0.0001; habitat: F_1,61_ = 7.354, p = 0.009). Body mass was strongly positively correlated to body size, but did not differ between the sexes or habitats when corrected for body length (ANCOVA: body length: F_1,118_ = 234.743, p<0.0001; sex: F_1,118_ = 0.720, p = 0.398; habitat: F_1,118_ = 0.597, p = 0.442; sex×habitat interaction: F_1,118_ = 1.559, p = 0.215).

## Discussion

We show that brains of Lake Mývatn male three-spine stickleback are substantially (ca. 23%) larger than those of females. This finding indicates that divergent natural and/or sexual selection can drive divergence in brain size not only between species [30], but also between the sexes within a species. The design of our study only allows us to speculate over the evolutionary reasons for the reported brain size dimorphism, but we propose that this brain size dimorphism is likely driven by the high cognitive demands of mate attraction and parental care in males. In stickleback, males construct elaborate nests and care for offspring alone [24], and during courtship perform elaborate displays based on visual ornament communication [24] – all behaviours that have been hypothesized to demand high cognitive ability [19]. Under the assumption that greater neural mass means greater information processing capacity [31], male stickleback likely benefit from increased brain mass. In support of this view, larger brains have also been shown to coincide with bower complexity in male bowerbirds [17], and with single parenting by females in cichlids [18] and carnivores [32]. In our study, we attribute the larger brains in males at least partly to the elaborate nests that males build and use as sexual display. Since nest complexity varies both among and within populations in stickleback [E.g. 33], further studies may reveal parallels to bower birds, where brain size and bower complexity are positively correlated [17]. It is interesting that with regards to the link between sexual selection and cognitive ability, it is entirely feasible that also the choosing sex (here the female) could be under strong selection for cognitive ability. Females must compare available males, their nests and courtship displays and also be able to store this information as they decide which male will sire their offspring [34]. However, our results suggest that the cognitive demands are higher in the sex under stronger sexual selection (here the male), or at least that the cognitive demands of choosing a mate are not sufficient to compensate for the demonstrated difference in brain size in three-spine stickleback. Future studies comparing species with different mating systems will be important to disentangle the effect of sexual selection on brain evolution from the perspective of both sexes.

However, the smaller brains in female stickleback could also be generated through directed selection or a plastic response of a decrease in brain size in females, for instance due to a trade-off between energetically expensive brain tissue and costly investment in fecundity [20]. Female sticklebacks invest heavily into egg production (the gonads may compose up to 40% of total body weight [24]), which may come at a cost to brain development. A similar pattern has previously been demonstrated in bats, where males trade testis mass against brain mass [21].

Interestingly, we also found that males from the lava habitat have larger brains than males from the mud habitat. If larger brains in males are due to the cognitive demands of uniparental brood care, the finding of larger brains in one of the morphs may reflect habitat specific differences in parental effort, cognitive demands or resource availability. Potential for differences arising due to variation in parental effort is supported by a recent laboratory study showing that the lava males perform more intense brood care (i.e. spend more time fanning the eggs [25]). Habitat-specific brain size divergence has been previously documented in the nine-spine stickleback [35], but our study is the first to document sex specific variation in brain size. We suggest that parental effort is a likely candidate for the selective force behind the observed brain size differences between males from the two habitats, since the numerous other ecological differences between the mud and lava habitats should be relatively similar for both sexes. A comparable pattern was also recently demonstrated in cichlid fish, where the females in species with uniparental female care had larger brains than females in species with biparental care [18]. However, our limited sample size of one population per habitat type does not allow us to generalize beyond the populations studied here. Future studies should therefore investigate whether the pattern holds across a larger sample of populations.

Our study used stickleback of two morphs from a single Icelandic lake, and it is too early to conclude that sexual dimorphism in brain size is a general feature of stickleback. However, although there is considerable variation in brood care intensity and nest building in different stickleback populations [24], males always court females and care for the eggs. Habitat specific differences in brain size may exist [35], [36], but as the relative differences in selective pressures between males and females are likely to be similar among populations, sexual brain size dimorphism should exist also in other populations. To our knowledge only one study has previously reported sexual dimorphism in stickleback brains. In 1921, Titschak compared four males and four “similar-sized” females of German sticklebacks, and reported that “all parts in the male brains were larger than the corresponding female ones”[37]. Future studies will need to investigate to what extent the brain size variation reflects plastic versus genetic differences, whether the observed brain size difference between the sexes actually translates into cognitive differences, and the extent of habitat specific sexual dimorphism. Also, as sex-specific, or even antagonistic selection pressures are common, future analyses on sexual dimorphism in brain size across taxa can shed light on the relationship between ecology and brain evolution. Ideally, such studies should target species, which differ in sex-bias (i.e. species with either male or female bias) of the concerted cognitive demands from parental care and sexual selection.
